# Gene Expression and Cardiometabolic Phenotypes of Vitamin D-Deficient Overweight and Obese Black Children

**DOI:** 10.3390/nu11092016

**Published:** 2019-08-28

**Authors:** Kumaravel Rajakumar, Qi Yan, Arshad T. Khalid, Eleanor Feingold, Abbe N. Vallejo, F. Yesim Demirci, M. Ilyas Kamboh

**Affiliations:** 1 Department of Pediatrics, UPMC Children’s Hospital of Pittsburgh, University of Pittsburgh, Pittsburgh, PA 15224, USA; 2 Division of Pediatric Pulmonary Medicine, UPMC Children’s Hospital of Pittsburgh, University of Pittsburgh, Pittsburgh, PA 15224, USA; 3 Department of Human Genetics, University of Pittsburgh, Pittsburgh, PA 15261, USA

**Keywords:** c-reactive protein, gene expression, leptin, endothelium, vitamin D deficiency

## Abstract

Associations between whole blood transcriptome and clinical phenotypes in vitamin D-deficient overweight and obese children can provide insight into the biological effects of vitamin D and obesity. We determined differentially expressed genes (DEGs) in relation to body mass index (BMI) in vitamin D-deficient black children with a BMI ≥ 85th percentile and ascertained the cardiometabolic phenotypes associated with the DEGs. We examined whole-blood transcriptome gene expression by RNA sequencing and cardiometabolic profiling in 41, 10- to 18-year-old children. We found 296 DEGs in association with BMI after adjusting for age, race, sex, and pubertal status. Cardiometabolic phenotypes associated with the BMI-related DEGs, after adjusting for age, sex, pubertal status, and %total body fat, were (i) flow-mediated dilation (marker of endothelial function), (ii) c-reactive protein (marker of inflammation), and (iii) leptin (adipocytokine). Canonical pathways of relevance for childhood obesity and its phenotypes that were significantly associated with the BMI-related DEGs affected immune cell function/inflammation, vascular health, metabolic function, and cell survival/death; several immune and inflammatory pathways overlapped across the three phenotypes. We have identified transcriptome-based biomarkers associated with BMI in vitamin D-deficient, overweight and obese black children. Modulating effects of vitamin D supplementation on these biomarkers and their related phenotypes need further exploration.

## 1. Introduction

Obesity is associated with a chronic inflammation, systemically and locally at fat deposits, induced by various inflammatory factors that are implicated in metabolic dysfunction [1,2,3]. There has been an increased prevalence of obesity and obesity-related disorders worldwide over the past four decades [4,5]. Childhood obesity tends to be a precursor to adulthood obesity and many of the obesity-associated adult comorbidities may begin at a young age. Therefore, understanding the molecular and genetic signatures of obesity in children is essential for mitigating the risk, and for improving the management, of obesity-related pathophysiology.

A higher burden of obesity is also associated with increased incidence of vitamin D deficiency [6,7,8] in adult and pediatric populations [9]. Vitamin D is essential for calcium homeostasis and bone health, and its role in treatment and prevention of rickets was established nearly 100 years ago. Our understanding of the function of vitamin D biology has been greatly expanded. Its effects are pleiotropic and extend beyond bone health and include immune modulatory activities [10,11]. Widespread tissue distributions of vitamin D receptor (VDR) and 1-α-hydroxylase, the enzyme that coverts 25-hydroxyvitamin D [25(OH)D] to its bioactive form, 1,25(OH)_2_D, in cells of various tissues, are key factors in the paracrine and autocrine effects of vitamin D. Interaction between bioactive vitamin D [1,25(OH)_2_D] and VDR elicits expression of a wide array of gene products that have many beneficial health effects [12,13].

Studies of whole blood gene expression profiling of vitamin D-deficient children and examination of the phenotypes associated with differentially expressed genes may provide insight into the pleiotropic regulatory effects of vitamin D in children. Data regarding in vivo gene expression profiles in vitamin D-deficient children are limited. In type-2 diabetic adults [14,15] and in adult-aged activity-discordant twins [16], metabolic disease phenotypes are profoundly influenced by genes in the immune and inflammatory pathways. Given the role of vitamin D as an immunologic and inflammatory mediator [17,18,19,20], understanding its impact on gene expression, especially as it relates to cardiometabolism, is warranted in order to understand its comprehensive biological effects. To broaden our current understanding of the biological determinants of obesity-associated cardiometabolic risk factors, we sought to (i) characterize differentially expressed genes (DEGs) in relation to body mass index (BMI) in vitamin D-deficient, overweight and obese black children, (ii) determine the cardiometabolic phenotypes associated with the BMI-related DEGs, and (iii) identify canonical gene expression pathways relevant to the determined phenotypes.

## 2. Materials and Methods

### 2.1. Study Design and Participants

We conducted a cross-sectional study in 43 (female 27, black 41) otherwise healthy, vitamin D-deficient (serum 25-hydroxyvitamin D < 20 ng/mL), overweight or obese (BMI ≥ 85th percentile), 10- to 18-year-old Pittsburgh-area children. They were recruited between 2013 and 2018 and enrolled in a randomized controlled clinical trial designed to examine the vascular and metabolic health benefits of vitamin D_3_ supplementation (ClinicalTrials.gov ID: NCT01797302). Participants were mainly recruited from the Primary Care Center of University of Pittsburgh Medical Center (UPMC) Children’s Hospital of Pittsburgh. Advertisements placed through UPMC Children’s Hospital of Pittsburgh, the University of Pittsburgh Clinical and Translational Science Institute (CTSI), and Pediatric PittNet (affiliated pediatric office-based research network) enhanced our recruitment effort to reach potential participants in the Greater Pittsburgh area. Participants’ screening visits were completed at the Primary Care Center and all their subsequent study visits were conducted at UPMC Montefiore Clinical and Translational Research Center. The study was approved by the Human Research Protection Office of the University of Pittsburgh. Informed parental consent and participant assent were obtained prior to participation. Participants’ race and ethnicity was specified by the parents or by participant self-identification if they were 18 years of age.

### 2.2. Study Measurements

Baseline study measurements from the screening/randomization visit were used for this analysis. Participants fasted for 8–10 h prior to their visit. Vascular health assessments, serum parathyroid hormone (PTH), glucose, and lipid battery, and plasma c-reactive protein (CRP), interleukin (IL)-6, leptin, and adiponectin were collected in the fasting state.

Anthropometry. Participants’ weight and height were measured three times using a digital weighing scale (Model 758C, Detecto Digital Weighing Scale, Webb City, MO, USA) and a stadiometer (Model 242, Seca Digital Stadiometer, Hamburg, Germany), respectively. Subjects shed easily-removable clothing (e.g., jackets, sweatshirts, shoes, etc.), but maintained a layer of clothing, when measured. Measurements were averaged, and BMI was calculated. Waist circumference was measured three times between the lowest rib and iliac crest during minimal respiration while standing, and then averaged. Waist-to-height ratio was then calculated. Waist circumference was obtained directly against the skin, in the absence of a clothing layer around the waist.

Body composition assessment. Dual-energy x-ray absorptiometry whole-body scan was performed using a Discovery Densitometer (Hologic Inc., Bedford, MA, USA) and percent of total body fat was ascertained. Subjects removed their outer clothing and wore a hospital gown for testing; clothing with embedded metal accessories and jewelry were removed prior the scan.

Biochemical measurements. Serum 25(OH)D, PTH, glucose, and lipid battery were assayed at the UPMC Clinical Chemistry laboratory. Serum 25(OH)D was assayed by a liquid chromatography–tandem mass spectrophotometry system as previously described [21]. The interassay coefficient of variation of this assay at low concentration (<10 ng/mL) is <13% and at mid- (10–60 ng/mL) and high-range (60–100 ng/mL) is <10%. PTH was measured by a chemiluminescent immunoassay with an overall interassay coefficient of variation of <5.5%, and the imprecision was similar throughout the assay range from 3 to 2500 pg/mL. Plasma adiponectin and leptin were analyzed using multiplexed assays as described previously [22,23]. Plasma high sensitivity CRP and interleukin-6 (IL-6) were measured through a multiplex technique using a Luminex system according to established protocols [24].

Vascular health assessments. We measured systemic blood pressure (BP) after subjects rested in the supine position for 10 min. Readings were taken using an automated digital oscillometric monitor (CONTEC08A, Contec Healthcare, Spartansburg, SC, USA) with an auto-inflate cuff (22–32 cm); measurements were taken three times and averaged. In a small subset of extremely obese subjects, Welch Allyn Connex^®^ Spot Monitor automated digital oscillometric device with GE Critikon Blood Pressure Cuff Sensa-Cuf 2491 Large Adult Long (cuff: 31–40 cm) was used instead to obtain BP measurements. 

Vascular health assessments included examination of arterial stiffness by assessment of carotid-femoral pulse wave velocity (PWV), pulse wave analysis (PWA), and endothelial function by percent change of brachial artery flow-mediated vasodilation (FMD%). Participants fasted for 8–10 h before testing. Using arterial tonometry (SphygmoCor CVMS V9, CPVH System, Model EM3, AtCor Medical, Sydney, Australia), we non-invasively measured PWV and PWA characteristics including aortic augmentation index adjusted to a heart rate of 75 beats-per-minute (AIx-75) and central (aortic) blood pressure. Higher values of PWV and AIx-75 indicated a greater degree of arterial stiffness.

Brachial artery flow-mediated dilation was assessed using high-resolution ultrasound machine (GE, Vivid 7, GE Health care, Milwaukee, WI, USA), equipped with a 9-L linear transducer preset to a dedicated vascular scanning protocol, which was used to measure the brachial artery diameter. After measuring the baseline luminal diameter, the brachial arterial flow was occluded for five minutes at the upper forearm using a 5-cm-wide occlusive cuff (SC5, Hokanson, Bellevue, WA, USA) inflated to 50 mmHg above the systolic BP or 200 mmHg, whichever was greater, by a rapid release sphygmomanometer (DS400, Hokanson, Bellevue, WA, USA). Post-cuff release diameter measurements during the reactive hyperemic phase (obtained at 60, 120, and 180 s) were used to calculate FMD%.

RNA sequencing. We analyzed the whole transcriptome broad gene expression by RNA sequencing using viable PAXgene blood RNA samples. High quality RNA (RNA Integrity Number >6) isolated from PAXgene tubes (500 ng) were used to generate sequencing libraries using the Illumina TruSeq Stranded Total RNA with Ribo-Zero Human/Mouse/Rat (H/M/R) kit. Ribosomal RNA (rRNA) and globin RNA in the sample were depleted by selective binding to biotinylated probes and the probe-bound rRNA and globin RNA were captured by magnetic beads and removed. This process maximized the percentage of uniquely mapped reads, including mRNA and non-coding RNA species. This kit typically generates ≥98% of uniquely mapped reads with accurate strand origin information. The RNA was fragmented prior to cDNA (complementary DNA) synthesis and was followed by adapter ligation. The libraries were then PCR amplified and purified using AMPure XP beads. Quality control (quantity, size and purity) of these libraries was performed using Agilent DNA Analysis ScreenTape on the 2200 TapeStation (Agilent Technologies, Santa Clara, CA, USA), a Qubit 2.0 Fluorometer (Life Technologies, Carlsbad, CA, USA), and a KAPA Library Quant kit (KAPA Biosystems, Wilmington, MA, USA). The libraries were normalized to 2 nM prior to sequencing. The created libraries were run on an Illumina NextSeq 500 sequencer using NextSeq 500 High Output Kit (150 cycles) (Illumina, Inc., San Diego, CA, USA), as per manufacturer’s protocol, in the Health Sciences Sequencing Core at the UPMC Children’s Hospital of Pittsburgh.

## 3. Statistical Analysis

### RNA-seq Processing and Data Analysis

Quality control for raw fastq files was performed using *FastQC* [25]. The low-quality reads and 3-prime adapters were trimmed with *Trim Galore!* [26]. The RNA sequence aligner, *STAR* [27], was used to align the trimmed reads to the reference human genome (hg19). Gene expression was subsequently quantified by counting the number of read fragments uniquely mapped to genes using *featureCounts* [28]. *DESeq2* was used to perform the statistical analyses and identify differentially expressed genes (DEGs) based on the raw counts [29]. This analysis used a linear model to examine the association between gene expression and BMI with adjustment for age, gender, race and pubertal status (Tanner stage). The significance cutoff was set at a false discovery rate (FDR) of <0.05.

We then used the significantly differentially expressed genes identified in the first step and tested their association, in black children only, with cardiometabolic variables: central and peripheral systolic and diastolic blood pressures; endothelial function (FMD%); arterial stiffness indices (PWV, AIx-75); serum total cholesterol, HDL, LDL, non-HDL cholesterol, triglycerides, and triglyceride-HDL ratio; plasma adiponectin, leptin, IL-6, and CRP. These analyses were adjusted for age, gender, pubertal status and percent total body fat. These were restricted only to black children because they represented the major race group of this cohort (*n* = 41, 95%).

The final step of analysis was to ask whether the genes whose expression is associated with each cardiometabolic variable are clustered into particular pathways, and whether those pathways are similar or different for the different cardiometabolic variables. To test pathways, we used the Ingenuity Pathway Analysis (IPA) software (application v. 463341M, content v. 42012434, Ingenuity Systems Inc., Redwood City, CA, USA). Statistical significance for this analysis was set at a *p*-value < 0.05. Significance was measured as the probability that our experimental gene set has the same genes as any given canonical pathway by random chance alone (by Right-tailed Fisher’s Exact Test). Lower p-values indicate a decreased probability of random association. We used these results to identify pathways that appeared across multiple phenotypes.

## 4. Results

We examined the association between gene expression and BMI in 43, 10- to 18-yr-old, overweight and obese, vitamin D-deficient children (mean age ± SD: 13.3 ± 2.2 years, BMI: 30 ± 5.6 kg/m^2^, 25(OH)D: 13.7 ± 4 ng/mL, obese 27, female 27, black 41). The demographic and cardiometabolic phenotype characteristics of all black children (*N* = 41) are shown in Table 1. The cardiometabolic phenotype characteristics that were associated with the BMI-related DEGs after adjustments for age, sex, pubertal status, and percent total body fat among black children were FMD% (a measure of endothelial function), leptin (an adipokine), and CRP (an inflammatory marker). BMI was positively associated with leptin (*r* = 0.378, *p* = 0.03, *n* = 33) and CRP (*r* = 0.412, *p* = 0.029, *n* = 28). No association was detected between BMI and serum concentrations of 25(OH)D (*r* = 0.11, *p* = 0.51, *n* = 41), or between BMI and FMD% (*r* = 0.19, *p* = 0.24, *n* = 40).

### 4.1. Differentially Expressed Genes Associated with BMI

A total of 296 genes were differentially expressed in association with BMI after adjusting for age, race, sex, and pubertal status among 26,364 transcripts that were sequenced and available for analysis (Table S1). The top 20 DEGs that were associated with BMI are listed in Table 2, and 18 of them had a positive association with BMI. Broadly, these genes regulate (i) immune and inflammatory activation (*LILA5, TLR5, EXOSC10, FCER1G, UBE2F, HCAR2, CD55, IL4R, and LAMTOR5*), (ii) general cell growth/signaling/differentiation (*ANXA3, S100A12, EXOSC10, S100A9, WDR46, and UBE2F)*, (iii) general cellular effector and energy production function (*EXOSC10, NDUFB2, PFKL, and SRPK*) (iv) vascular (*ANXA3*), metabolic (*SLC37A3 and DEGS1*), integumentary (*PXK*), and muscular (*MEF2A*) function.

### 4.2. Association between Cardiometabolic Phenotypes and BMI-Related Genes

**Figure 1:**
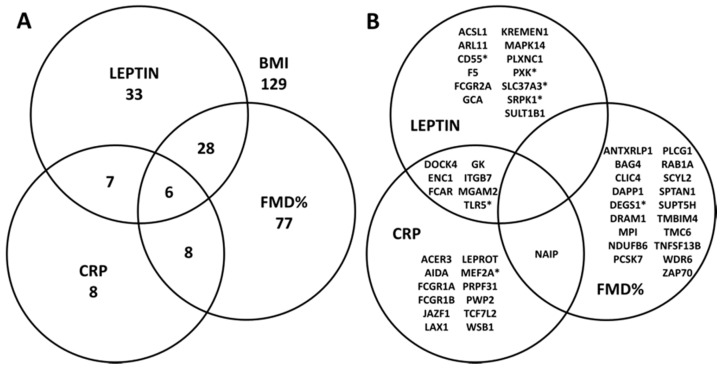
Overlap of significant differentially expressed genes by BMI-related cardiometabolic phenotypes. (**A**) Venn-diagram depicting overlapping frequency of all BMI-related significantly differentially expressed genes (*N* = 296) by each cardiometabolic phenotype; Of the 296 BMI-associated DEGs 129 were not associated with any of the three cardiometabolic phenotypes. (**B**) Venn-diagram representing the top 20 differentially expressed genes within each cardiometabolic phenotype; * The top 20 DEGs specific to BMI are indicated by asterisks symbol. BMI, body mass index; CRP, C-reactive protein; FMD%, brachial artery flow-mediated dilation percentage.

The number of BMI-related DEGs associated with FMD%, leptin, and CRP in the adjusted analyses were 119, 74 and 29, respectively (Figure 1A, Table S2). We further examined the functional relevance of the top 20 genes associated with the respective cardiometabolic phenotype.

BMI-related differentially expressed genes associated with FMD%. The top 20, out of 119, BMI-related DEGs associated with FMD% are shown in Figure 1B and Table S3. Eleven of these top 20 genes regulate a wide array of general cellular functions, including intracellular vesicular traffic (*RAB1A, PCSK7, and SCYL2*), autophagy (*DRAM1*), ionic homeostasis (*CLIC4 and TMBIM4*), cell cycle activity (*WDR6 and SPTAN1*), and apoptosis (*BAG4 and NAIP*). The remainder of the genes are regulators of immune cascades (*ZAP70, SUPT5H, DAPP1, BAG4 and TNFSF13B*) and metabolic effector functions (*NDUFB6, MPI, and DEGS1*). Nine of them were inversely associated.

BMI-related differentially expressed genes associated with leptin. The top 20, out of 74, BMI-related DEGs associated with leptin are listed in Figure 1B and Table S4. These genes regulate (i) cell signaling (*KREMEN1*), transcription regulation (*SRPK1*), motility (*PLXNC1*), and enzyme activity (*SULT1B1*), (ii) immune function (*TLR5, FCAR, MAPK14, PLXN1C, FCGR2A, CD55, ITGB7, and GCA*), (iii) metabolic effector function (*SLC37A3, ACSL1, and MGAM2*), and vascular function (*F5*). Only one of these was inversely associated.

BMI-related differentially expressed genes associated with CRP. The top 20, out of 29, BMI-related DEGs associated with CRP are enumerated in Figure 1B and Table S5. They regulate (i) innate immune signaling and inflammasome activity (*TLR5, ITGB7, FCGR1A, FCAR, FCGR1B, LAX1, and NAIP*), (ii) metabolic function (*MGAM2, TCF7L2, and GK*), (iii) muscle function (*MEF2A*), and (iv) cellular function (*WSB1, PWP2, LEPROT, and ACER3*). Four of these top 20 genes were inversely associated.

Overlap among BMI-related DEGs associated with FMD%, leptin, and CRP are shown in Figure 1. Of the 296 BMI-related DEGs, 129 were not associated with FMD%, CRP, or Leptin. Six genes (*C9orf72, GK, SLC16A6, TLR5, TNFSF12B, and WSB1*) were found to have a significant expression across all three cardiometabolic phenotypes (leptin, CRP, and FMD%). Leptin and FMD% represent the greatest overlap of expressed genes (*n* = 34) relative to all other cardiometabolic phenotypic pairings. Whereas, among the top 20 BMI-related DEGs associated with the three phenotypes, none of them overlapped across all three phenotypes. However, *NAIP* crossed over between FMD% and CRP; and *DOC4, ENC1, FCAR, GK, ITB7, MGAM2, TLR5* overlapped between leptin and CRP.

### 4.3. Canonical Pathways Associated with Significant Genes

From 66 IPA-identified canonical pathways that were significantly associated with BMI-related DEGs, 24 were of obvious functional relevance in the context of childhood obesity and its attendant risk phenotypes (Table 3). 

**Figure 2:**
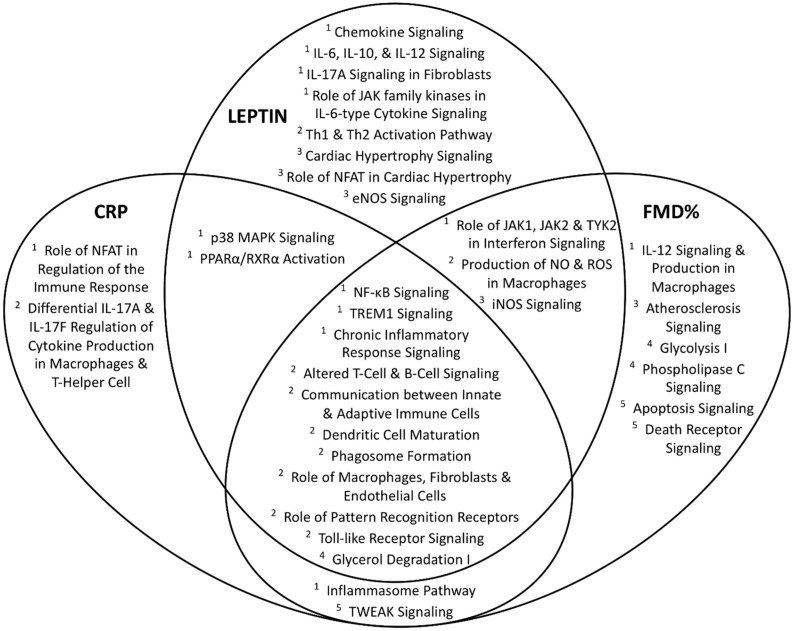
Overlap of significant IPA-derived canonical pathways by BMI-related cardiometabolic phenotypes. Venn-diagram depicting overlap of IPA-derived functional canonical pathways associated with each of the significant cardiometabolic phenotypes; pathway classifications are annotated as ^1^ inflammatory signaling, ^2^ immune cell function, ^3^ cardiovascular effect, ^4^ metabolic functions, and ^5^ cell survival/death. CRP, C-reactive protein; FMD%, brachial artery flow mediated dilation percentage; BMI, body mass index; IPA, Ingenuity Pathway Analysis

Most of these pathways affect immune function and inflammation. Phagosome formation, chronic inflammatory responses, IL-10, NF-κB, and TREM1 signaling represented the most significantly associated inflammatory pathways. TLR signaling, dendritic and macrophage cell function were the most significantly associated innate immune pathways. Several inflammatory and immune pathways overlapped among the three significant cardiometabolic phenotypes (FMD%, CRP, and leptin), which were discovered during the second phase analysis of cardiometabolic phenotypes associated with BMI-related DEGs (Figure 2).

Gene expression related to these phenotypes suggested increased expression of inflammatory signaling relevant to NF-κB, TREM1, and chronic inflammatory response. Immune-related pathways overlapping across all three phenotypes included altered T- and B-cell signaling, communication between innate and adaptive immune cells, dendritic cell maturation, phagosome formation, involvement of macrophages, fibroblasts, and endothelial cells, pattern recognition receptors, and toll-like receptor signaling. Cardiovascular pathways associated with leptin included cardiac hypertrophy signaling and eNOS signaling. The atherosclerosis signaling pathway was associated with FMD% (endothelial function). The iNOS signaling pathway was associated with both leptin and FMD%.

## 5. Discussion

Our findings indicate that only a small of number of genes (≈300) with diverse biological functions are differentially expressed in association with BMI in vitamin D-deficient overweight and obese black children. Endothelial function, CRP concentrations, and leptin concentrations were the only three cardiometabolic phenotypes that were associated with the BMI-associated genes. The overlap across the clusters of the top 20 BMI-related genes found in association with endothelial function, CRP concentrations, and leptin concentrations was minimal. However, there were a considerable number of overlapping functional pathways across these three significant phenotypes. Most of the overlapping pathways were relevant for immune function or mediation of inflammation. In addition, pathways of relevance for endothelial function, metabolism, and apoptosis were also noted. Obesity is associated with a chronic, underlying systemic inflammation; increased inflammation is an important driver of metabolic dysfunction. Our findings indicate that genes expressed for immune and inflammatory functions in vitamin D-deficient overweight and obese black children have overlapping effects on cardiometabolic risk phenotypes.

Obesity is associated with vitamin D deficiency [30]. The lack of association between 25(OH)D concentrations and BMI in our cohort can be explained by inclusion of only vitamin D-deficient children. Vitamin D deficiency is emerging as an independent cardiometabolic risk factor in children. In previous studies, we have shown a positive association between serum 25(OH)D concentrations and insulin sensitivity in black children [31], and an inverse association between serum 25(OH)D concentrations and HDL cholesterol in black and white American children [30]. Study of gene expression profiles in peripheral blood cells of vitamin D-deficient obese children can provide insight into genes that are affected by obesity and/or vitamin D status. In our cohort, some of the obesity-related genes were associated with leptin, an adipocytokine; CRP, an inflammatory marker; and FMD%, an index of endothelial function. Most of these genes influenced pathways of relevance for immune function and inflammation, which is consistent with the study of gene expression from peripheral blood cells. However, several of these genes were of relevance for cardiac health and endothelial function.

Obese children, in general, have higher concentrations of leptin and CRP and are vulnerable for endothelial dysfunction. The shared pathways between leptin, inflammatory markers, and endothelial function may help to explain cardiovascular dysfunction in obese populations. Previous experimentation on murine and canine models suggests that coronary arteries with the leptin receptor were associated with increased endothelial dysfunction in the presence of obese-level concentrations of leptin, but normal endothelial function in normal levels of leptin [32]. Obese levels of leptin contributed to the impairment of nitric oxide-mediated vasodilation. The pathways examined in our investigation showed similar functional overlap.

FMD is a validated, non-invasive measure of endothelial dysfunction. The nitric oxide-mediated response on the endothelium [33], derived from ischemia in the peripheral artery, strongly predicts cardiovascular dysfunction in adults [34]. Inflammation and oxidative stress play a key role in the development of cardiovascular pathophysiology, especially in the early stages of atherosclerosis [35,36,37]. Abnormal levels of several pro-inflammatory markers (CRP, leptin, TNF-a, IL6, and adiponectin) have been associated with poorer cardiovascular outcomes in adult and obese children. Our findings suggest a potential link between inflammatory markers and a marker of endothelial dysfunction by highlighting the specific canonical pathways that may be used. The overlap between leptin and FMD, especially as it relates to cardiovascular-related functional pathways, supports the substantial, potentially direct role that leptin may play in endothelial dysfunction and subsequent cardiometabolic pathology.

Our investigation was limited by the sample size and diversity of the cohort and a more robust sample is necessary to build on the findings of this study. A larger and a more diverse sample with inclusion of children across the BMI spectrum and vitamin D status is necessary to isolate the effects of race, adiposity, and vitamin D status on gene expression. The smaller sample size warranted the inclusion of genes for pathway analysis using a more lenient threshold based on *p*-values only; fold change thresholds could not be implemented. Furthermore, the methodology used is based on a single baseline timepoint—differential expression is relative to the baseline value of each phenotype. Evaluating differential expression across multiple timepoints, before and after the vitamin D intervention, is necessary for a more nuanced understanding of the role of vitamin D.

The likely contribution of persistent, low-grade inflammation to metabolic and cardiovascular pathways was particularly relevant. An over-representation of immune/inflammatory signaling genes was evident. This may be due to the use of peripheral blood cell (PBC) samples. PBCs have been proposed as a useful tissue source to study cardiovascular and acute coronary disease [38,39] and have been used to characterize immune function [40,41]. Furthermore, PBCs represent a comparatively non-invasive, convenient means to collect relevant obesity-related cell types for study. Invasively procuring adipose or muscle tissue samples introduces ethical challenges and complexities, especially in the context of pediatric subjects, where cardiometabolic disease may still be in its early, subclinical stages. In a transcriptome analysis of PBCs of 32 young (4.7 to 8 years of age), normal-weight (*N* = 17, male = 9) and overweight (*N* = 15, male = 7), Spanish children, a total of 1077 genes were differentially expressed between overweight and normal-weight children [42]. The biological functions represented by these DEGs were concordant with the functional relevance of our top 20 BMI-associated genes, and affected (1) transcription/translation machinery, (2) cell turnover and signaling, (3) immune system, and (4) and a wide-array of metabolic functions. In a gene expression analysis of subcutaneous adipose tissue obtained from obese (*N* = 7) and lean (*N* = 8) children, a total of 199 genes were differentially expressed in obese children compared to non-obese children [43]. Of those genes, 79 were upregulated and 120 were downregulated. Like our findings, the upregulated DEGs in obese children were enriched in pathways associated with the immune system.

## 6. Conclusions

Only a limited number of genes are differentially expressed in association with BMI in the PBCs of vitamin D-deficient overweight and obese children. Furthermore, those genes were related to endothelial function, CRP and leptin concentrations, and mediated several immune and inflammatory signaling pathways, with considerable overlap when examined across the three phenotypes. Future research needs to address the biological relevance related to overlapping vs. non-overlapping genes and pathways. Although a few genes in a pathway may seem to be over- or under-expressed relative to the baseline, it remains necessary to distinguish what subjects’ traits account for the differential expression. Furthermore, the modulating effects of vitamin D supplementation on DEGs on a longitudinal scale and by varying concentrations of supplementation need further elucidation. Integrating the transcriptomic data with GWAS-derived genotypic information may further contribute to understandings of the associations between whole blood transcriptome and clinical phenotypes of vitamin D deficiency and/or obesity.

## Figures and Tables

**Table 1:** Demographic and Cardiometabolic Phenotype Characteristics in Black Children.

Characteristics	*N* = 41
***Demographic***	
Female	26 (63)
Non-Hispanic	40 (98)
Age, yrs	13.2 ± 2.0
***Anthropometrics***	
Weight, kg	80.6 ± 19.3
Height, cm	163.3 ± 10.9
BMI, kg/m^2^	30.0 ± 5.7
BMI percentile	95.8 ± 4.0
Waist circumference, cm	89.9 ± 14.4
Waist-to-height ratio	0.55 ± 0.09
Percent total body fat	32.4 ± 8.4
***Weight classification***	
Overweight (BMI 85th to < 95th %tile)	16 (39)
Obese (BMI ≥ 95th %tile)	25 (61)
***Pubertal status, Tanner Stage***	
I	2 (5)
II	3 (7)
III	6 (15)
IV	17 (41)
V	13 (32)
***Laboratory data***	
25(OH)D, ng/mL	13.7 ± 4.1
PTH, pg/mL	48.1 ± 20.5
Total cholesterol, mg/dL	152.9 ± 24.6
LDL cholesterol, mg/dL	91.3 ± 23.9
HDL cholesterol, mg/dL	46.5 ± 9.8
Triglycerides, mg/dL	75.3 ± 25.1
Non-HDL cholesterol, mg/dL	106.4 ± 25.1
Triglyceride-HDL-ratio	1.7 ± 0.7
Leptin, ng/mL	18.4 ± 9.9
Adiponectin, ng/mL	13.2 ± 10
CRP, pg/mL	3028 ± 5546
Interleukin-6, pg/mL	6.9 ± 13.1
***Vascular health data***	
Baseline brachial artery diameter, cm	0.32 ± 0.05
FMD%	7.32 ± 5.4
PWV, m/sec	4.7 ± 0.7
AIx@75bpm	2.83 ± 11.2
Central systolic BP, mm Hg	98.1 ± 9.1
Central diastolic BP, mm Hg	68.3 ± 7.6
Systemic systolic BP, mm Hg	115.1 ± 11.1
Systemic diastolic BP, mm Hg	67.5 ± 7.3

Data shown as number (percentage) or mean ± SD, unless stated otherwise Missing data, *n*: PTH, 1; Leptin, 7; Adiponectin,7; CRP, 13; IL-6, 13; FMD%, 1; PWV, 2.

**Table 2:** Top 20 Differentially Expressed Genes Associated with BMI.

Gene	Name	Category	FDR *
*LILRA5*	leukocyte immunoglobulin like receptor A5	immune function (pro-inflammatory)	1.37 × 10^−3^
*ANXA3*	annexin A3	general cell growth/signaling vascular effects (anti-coagulation)	2.73 × 10^−3^
*PXK*	PX domain containing serine/threonine kinase like	integumentary effector	2.73 × 10^−3^
*S100A12*	S100 calcium binding protein A12	general cell growth/differentiation innate immune sensor (innate sensor, anti-bacterial)	2.73 × 10^−3^
*SLC37A3*	solute carrier family 37 member 3	potential metabolic effector (regulator of adipose tissue)	2.73 × 10^−3^
*TLR5*	toll like receptor 5	innate immune signaling	2.73 × 10^−3^
*EXOSC10*	exosome component 10	general cellular effector (RNA degradation) immune function (Ig class-switching, Ig extracellular trafficking)	8.22 × 10^−3^
*S100A9*	S100 calcium binding protein A9	general cell growth/differentiation innate immune sensor (anti-bacterial/fungal)	8.22 × 10^−3^
*WDR46*	WD repeat domain 46	general cellular function (nucleolar scaffolding, granule localization)	8.22 × 10^−3^
*DEGS1*	delta 4-desaturase, sphingolipid 1	metabolic effector (fatty acid desaturation)	1.02 × 10^−2^
*FCER1G*	Fc receptor for IgE	immune function (hypersensitivity)	1.05 × 10^−2^
*MEF2A*	myocyte enhancer factor 2A	muscular effector	1.05 × 10^−2^
*NDUFB2*	NADH:ubiquinone oxidoreductase subunit B2	general cellular energy production (electron transport system)	1.05 × 10^−2^
*UBE2F*	ubiquitin conjugating enzyme E2 F (putative)	general cellular function (cell cycle, protein folding)	1.05 × 10^−2^
*HCAR2*	hydroxycarboxylic acid receptor 2	innate immune function (neutrophil apoptosis activator)	1.15 × 10^−2^
*PFKL*	phosphofructokinase, liver type	general cellular energy production (glycolysis in liver)	1.15 × 10^−2^
*SRPK1*	SRSF protein kinase 1	general cell transcriptional regulation	1.15 × 10^−2^
*CD55*	cluster of differentiation 55	immune function (regulator of complement-driven cellular damage)	1.19 × 10^−2^
*IL4R*	interleukin 4 receptor	immune cell signaling	1.19 × 10^−2^
*LAMTOR5*	late endosomal/lysosomal adaptor, MAPK and MTOR activator	endosome formation, intracellular signaling	1.19 × 10^−2^

* Adjusted for age, gender, race, and pubertal status.

**Table 3:** Functionally relevant BMI-associated biological pathways (*n* = 24).

Pathways	*p*-Value
***Inflammatory Signaling***	
Phagosome Formation	1.38 × 10^−7^
Chronic Inflammatory Syndrome	1.62 × 10^−6^
IL-10 Signaling	3.16 × 10^−6^
NF-κB Signaling	2.69 × 10^−5^
TREM1 Signaling	5.50 × 10^−5^
Altered T-Cell & B-Cell Signaling	1.74 × 10^−4^
Role of PKR in Interferon Induction	1.86 × 10^−4^
Role of NFAT in Regulation of the Immune Response	7.76 × 10^−4^
Inflammasome Pathway	2.14 × 10^−3^
PPARα/RXRα Activation	2.57 × 10^−3^
p38 MAPK Signaling	4.90 × 10^−3^
IL-6 Signaling	2.63 × 10^−2^
Role of JAK family kinases in IL-6-type Cytokine Signaling	4.17 × 10^−2^
***Immune Cell Function***	
Toll-like Receptor Signaling	6.61 × 10^−6^
Role of Macrophages, Fibroblasts & Endothelial Cells	1.62 × 10^−5^
Dendritic Cell Maturation	4.79 × 10^−5^
Communication between Innate & Adaptive Immune Cells	2.29 × 10^−4^
Th1 & Th2 Activation Pathway	3.02 × 10^−3^
***Cardiovascular Effect***	
Cardiac Hypertrophy Signaling	1.26 × 10^−2^
iNOS Signaling	2.09 × 10^−2^
***Metabolic Functions***	
Phospholipase C Signaling	3.39 × 10^−4^
Glycolysis I	4.47 × 10^−2^
***Cell Survival/Death***	
TWEAK Signaling	1.05 × 10^−2^
Apoptosis Signaling	3.02 × 10^−2^

## Supplementary Materials

The following are available online at https://www.mdpi.com/2072-6643/11/9/2016/s1, Table S1: All significant differentially expressed genes associated with BMI; Table S2: All significant BMI-related differentially expressed genes associated with cardiometabolic phenotypes; Table S3: Top 20 differentially expressed genes associated with FMD%; Table S4: Top 20 differentially expressed genes associated with leptin; Table S5: Top 20 differentially expressed genes associated with CRP.
