# Supplementary material for: Gene Expression and Cardiometabolic Phenotypes of Vitamin D-Deficient Overweight and Obese Black Children
Source: Nutrients. 2019 Aug 28;11(9):2016. doi: 10.3390/nu11092016 (PMC6770908; doi:10.3390/nu11092016)
Supplement: Supplementary file 1 [file nutrients-11-02016-s001.pdf]

Table S1: All significant differentially expressed genes associated with BMI					
**Gene**	**Base Mean**	**Beta**	**Stat**	**P value**	**FDR***
LILRA5	1107.3753	0.0518	5.2554	1.477E-07	1.368E-03
S100A12	350.9780	0.0472	4.7951	1.626E-06	2.726E-03
TLR5	250.5398	0.0458	4.8389	1.305E-06	2.726E-03
PXK	327.8884	0.0295	4.7784	1.767E-06	2.726E-03
ANXA3	238.4919	0.0469	4.7937	1.637E-06	2.726E-03
SLC37A3	302.7028	0.0412	4.8988	9.641E-07	2.726E-03
EXOSC10	386.4947	-0.0233	-4.4663	7.957E-06	8.219E-03
S100A9	17741.9403	0.0438	4.4978	6.867E-06	8.219E-03
WDR46	197.7187	-0.0251	-4.4654	7.990E-06	8.219E-03
DEGS1	473.1677	0.0267	4.3964	1.101E-05	1.019E-02
FCER1G	472.5858	0.0338	4.3156	1.592E-05	1.053E-02
MEF2A	711.9469	0.0282	4.3288	1.499E-05	1.053E-02
UBE2F	178.9889	0.0278	4.3446	1.395E-05	1.053E-02
NDUFB2	92.1442	0.0334	4.3235	1.536E-05	1.053E-02
HCAR2	252.0360	0.0408	4.2518	2.120E-05	1.155E-02
PFKL	521.2294	-0.0285	-4.2779	1.887E-05	1.155E-02
SRPK1	703.0786	0.0361	4.2549	2.092E-05	1.155E-02
LAMTOR5	167.1592	0.0314	4.2014	2.653E-05	1.193E-02
CD55	2030.1914	0.0345	4.2007	2.661E-05	1.193E-02
IL4R	1504.4016	0.0298	4.1871	2.825E-05	1.193E-02
GYG1	164.5025	0.0334	4.1863	2.835E-05	1.193E-02
CYSTM1	159.3210	0.0407	4.2283	2.355E-05	1.193E-02
PROK2	708.4841	0.0407	4.1150	3.872E-05	1.556E-02
GPR160	194.0010	0.0356	4.0993	4.144E-05	1.556E-02
DOCK4	434.7192	0.0396	4.0960	4.203E-05	1.556E-02
CLIC1	1924.7494	0.0340	4.0652	4.799E-05	1.709E-02
RGL4	143.3448	0.0365	4.0456	5.219E-05	1.726E-02
GK	1056.5865	0.0387	4.0483	5.159E-05	1.726E-02
GOLPH3L	173.0063	0.0230	4.0142	5.965E-05	1.765E-02
F5	508.5471	0.0379	3.9918	6.557E-05	1.765E-02
ADM	261.3851	0.0394	4.0063	6.166E-05	1.765E-02
TMBIM4	815.0141	0.0318	3.9920	6.552E-05	1.765E-02
LAPTM4A	303.3148	0.0262	4.0163	5.911E-05	1.765E-02
IL1B	312.2155	0.0372	4.0023	6.273E-05	1.765E-02
TOMM6	205.0291	0.0259	3.9876	6.673E-05	1.765E-02
CNTROB	69.4692	-0.0308	-3.9721	7.124E-05	1.832E-02
ACTR10	287.1397	0.0258	3.9445	7.997E-05	1.920E-02
LETM1	204.4686	-0.0254	-3.9418	8.088E-05	1.920E-02
DYNLT1	251.0599	0.0312	3.9477	7.890E-05	1.920E-02
LARS2	210.3644	-0.0205	-3.9331	8.386E-05	1.941E-02
AMN1	148.4554	0.0316	3.9237	8.720E-05	1.947E-02
AQP9	3468.4775	0.0370	3.9206	8.831E-05	1.947E-02
ABCF3	224.2546	-0.0242	-3.9075	9.325E-05	1.975E-02
SERPINB1	1067.4159	0.0324	3.9060	9.385E-05	1.975E-02
SRGN	7524.5637	0.0373	3.8818	1.037E-04	2.000E-02
ESYT1	1151.9288	-0.0265	-3.8947	9.834E-05	2.000E-02
DDX24	765.3892	-0.0255	-3.8845	1.026E-04	2.000E-02
SUN2	2345.1515	-0.0227	-3.8858	1.020E-04	2.000E-02

DRAM1	132.6963	0.0322	3.8578	1.144E-04	2.025E-02
DUSP3	327.5436	0.0263	3.8629	1.121E-04	2.025E-02
FCAR	236.8464	0.0363	3.8660	1.106E-04	2.025E-02
NFKBIZ	1030.2855	0.0294	3.8611	1.129E-04	2.025E-02
MCCC1	210.6080	-0.0203	-3.8525	1.169E-04	2.025E-02
ENC1	231.8850	0.0373	3.8460	1.200E-04	2.025E-02
AQP3	100.4797	-0.0338	-3.8455	1.203E-04	2.025E-02
OSER1	489.4658	0.0263	3.8377	1.242E-04	2.054E-02
SULT1B1	304.3844	0.0371	3.8154	1.360E-04	2.209E-02
POLG	736.4064	-0.0182	-3.8101	1.389E-04	2.217E-02
TMEM70	130.5992	0.0254	3.8036	1.426E-04	2.238E-02
HCAR3	319.4549	0.0369	3.7910	1.500E-04	2.315E-02
METTL9	964.0059	0.0309	3.7865	1.528E-04	2.319E-02
VNN2	4730.1431	0.0372	3.7793	1.573E-04	2.349E-02
VCPKMT	227.1126	0.0304	3.7699	1.633E-04	2.400E-02
PBXIP1	1642.5602	-0.0244	-3.7652	1.664E-04	2.407E-02
TCF7L2	86.2305	0.0343	3.7582	1.711E-04	2.437E-02
LNX2	204.1792	0.0193	3.7447	1.806E-04	2.533E-02
WSB1	1208.0313	0.0266	3.7385	1.851E-04	2.558E-02
ATP5L	316.8534	0.0245	3.7292	1.921E-04	2.616E-02
CARKD	184.2031	-0.0243	-3.7170	2.016E-04	2.705E-02
NOP10	338.0546	0.0300	3.7019	2.140E-04	2.714E-02
MPI	138.2064	-0.0233	-3.7046	2.117E-04	2.714E-02
PIP4K2B	485.6790	-0.0200	-3.7075	2.093E-04	2.714E-02
IAH1	114.5021	0.0189	3.7038	2.124E-04	2.714E-02
PLCL1	177.4395	-0.0301	-3.6973	2.179E-04	2.726E-02
PANK4	250.6233	-0.0181	-3.6888	2.253E-04	2.739E-02
FCGR1B	110.0519	0.0364	3.6877	2.263E-04	2.739E-02
CEP128	148.5417	-0.0265	-3.6730	2.397E-04	2.739E-02
SRP68	196.6692	-0.0220	-3.6816	2.317E-04	2.739E-02
IL18RAP	724.2369	0.0356	3.6789	2.342E-04	2.739E-02
XPC	812.3270	-0.0185	-3.6674	2.451E-04	2.739E-02
TRIM35	332.0173	-0.0192	-3.6669	2.455E-04	2.739E-02
C9orf72	1705.3052	0.0357	3.6786	2.345E-04	2.739E-02
NDUFB6	136.9564	0.0284	3.6732	2.395E-04	2.739E-02
PRPF31	194.7920	-0.0235	-3.6518	2.604E-04	2.870E-02
EIF2B5	356.5932	-0.0188	-3.6465	2.658E-04	2.895E-02
LYZ	27128.9185	0.0271	3.6151	3.003E-04	3.031E-02
TMCO3	355.8479	0.0257	3.6081	3.085E-04	3.031E-02
TEFM	76.5978	0.0254	3.6104	3.057E-04	3.031E-02
AATF	369.6660	-0.0191	-3.6256	2.883E-04	3.031E-02
RNFT1	205.5662	0.0243	3.6281	2.856E-04	3.031E-02
POLRMT	148.9188	-0.0265	-3.6107	3.053E-04	3.031E-02
SUPT5H	331.8541	-0.0277	-3.6186	2.961E-04	3.031E-02
ZAP70	868.2358	-0.0253	-3.6090	3.074E-04	3.031E-02
WDR6	499.7117	-0.0280	-3.6195	2.951E-04	3.031E-02
TMEM165	514.2599	0.0286	3.6059	3.110E-04	3.031E-02
RIT1	634.0737	0.0260	3.5956	3.237E-04	3.118E-02
EIF4G2	2870.0547	0.0153	3.5932	3.267E-04	3.118E-02
CD48	1709.9768	0.0285	3.5830	3.397E-04	3.145E-02

CISD2	340.2705	0.0353	3.5847	3.375E-04	3.145E-02
MAPK14	2219.3924	0.0285	3.5864	3.352E-04	3.145E-02
REL	951.7885	0.0198	3.5730	3.529E-04	3.235E-02
RPN2	814.4438	-0.0190	-3.5677	3.601E-04	3.237E-02
DCP1A	341.5247	0.0154	3.5699	3.572E-04	3.237E-02
FTSJ3	248.0600	-0.0218	-3.5608	3.697E-04	3.291E-02
CEPT1	496.4758	0.0243	3.5572	3.749E-04	3.304E-02
ZNF438	222.5404	0.0327	3.5523	3.819E-04	3.304E-02
CCNDBP1	1720.9088	0.0297	3.5498	3.855E-04	3.304E-02
NAIP	649.1322	0.0338	3.5503	3.849E-04	3.304E-02
MS4A6A	878.4254	0.0297	3.5386	4.023E-04	3.330E-02
ANKZF1	468.0213	-0.0224	-3.5360	4.063E-04	3.330E-02
PWP2	238.2342	-0.0241	-3.5436	3.948E-04	3.330E-02
TNIK	582.0221	-0.0225	-3.5359	4.064E-04	3.330E-02
SURF6	195.1848	-0.0184	-3.5418	3.974E-04	3.330E-02
TMCO4	100.7364	-0.0285	-3.5228	4.270E-04	3.336E-02
NIPAL3	570.8017	-0.0249	-3.5196	4.323E-04	3.336E-02
LEPROT	436.9305	0.0262	3.5120	4.447E-04	3.336E-02
CD58	368.1696	0.0281	3.5230	4.268E-04	3.336E-02
SSR2	725.2117	0.0192	3.5151	4.396E-04	3.336E-02
DCPS	93.9230	-0.0240	-3.5274	4.196E-04	3.336E-02
ITGB7	707.3212	-0.0273	-3.5165	4.372E-04	3.336E-02
CHD6	1103.0903	-0.0195	-3.5191	4.330E-04	3.336E-02
TAB2	1124.6487	0.0218	3.5086	4.504E-04	3.336E-02
JAZF1	756.7697	0.0348	3.5279	4.189E-04	3.336E-02
NAMPT	12869.8969	0.0344	3.5107	4.470E-04	3.336E-02
MSL3	583.5025	0.0211	3.5291	4.170E-04	3.336E-02
ST3GAL4	135.1301	0.0330	3.5005	4.643E-04	3.359E-02
RASA3	1135.6895	-0.0258	-3.5009	4.637E-04	3.359E-02
CCDC117	385.1478	0.0184	3.5034	4.593E-04	3.359E-02
THYN1	147.7901	-0.0171	-3.4889	4.850E-04	3.428E-02
COX7A2L	297.8845	0.0269	3.4926	4.784E-04	3.428E-02
COPG1	614.0191	-0.0200	-3.4905	4.821E-04	3.428E-02
UQCRQ	118.2813	0.0288	3.4828	4.962E-04	3.454E-02
EHMT2	154.9362	-0.0281	-3.4835	4.949E-04	3.454E-02
EBLN2	104.7807	0.0240	3.4774	5.062E-04	3.471E-02
SEC62	1677.5974	0.0331	3.4790	5.033E-04	3.471E-02
DPY19L3	326.8875	0.0276	3.4752	5.104E-04	3.475E-02
PFKP	247.6779	-0.0237	-3.4650	5.303E-04	3.527E-02
NMT2	308.9009	-0.0238	-3.4611	5.380E-04	3.527E-02
ARL11	499.9333	0.0277	3.4520	5.564E-04	3.527E-02
TMEM260	448.3487	0.0235	3.4535	5.534E-04	3.527E-02
GEMIN4	156.4868	-0.0236	-3.4515	5.574E-04	3.527E-02
DHRS13	172.0298	0.0322	3.4503	5.600E-04	3.527E-02
SOCS3	326.5798	0.0339	3.4624	5.353E-04	3.527E-02
KCMF1	645.3323	0.0163	3.4610	5.383E-04	3.527E-02
LSM1	91.4316	0.0281	3.4582	5.439E-04	3.527E-02
NFIL3	245.1954	0.0318	3.4505	5.595E-04	3.527E-02
GOLGA2	264.2292	-0.0200	-3.4594	5.414E-04	3.527E-02
APEH	363.8007	-0.0162	-3.4466	5.677E-04	3.528E-02

DAPP1	1240.6290	0.0278	3.4468	5.672E-04	3.528E-02
TP53BP1	440.5724	-0.0202	-3.4403	5.811E-04	3.587E-02
KIAA0226L	801.0305	0.0270	3.4312	6.010E-04	3.608E-02
THRA	81.8461	-0.0287	-3.4313	6.006E-04	3.608E-02
CACTIN	135.5503	-0.0248	-3.4303	6.030E-04	3.608E-02
KPNA4	1201.2477	0.0174	3.4313	6.007E-04	3.608E-02
AKIRIN2	176.3078	0.0250	3.4298	6.041E-04	3.608E-02
TMEM59	1037.5548	0.0241	3.4013	6.707E-04	3.692E-02
ATP8B2	1107.0191	-0.0256	-3.4150	6.379E-04	3.692E-02
CHD4	1258.7637	-0.0230	-3.4041	6.638E-04	3.692E-02
SCYL2	724.9894	0.0242	3.4071	6.565E-04	3.692E-02
TNFSF13B	893.3545	0.0332	3.4157	6.362E-04	3.692E-02
SDR39U1	116.9363	-0.0196	-3.4156	6.364E-04	3.692E-02
LYRM1	153.8780	0.0217	3.4036	6.649E-04	3.692E-02
ELAC2	377.0126	-0.0185	-3.4008	6.719E-04	3.692E-02
SUPT4H1	441.5488	0.0239	3.4062	6.587E-04	3.692E-02
CXXC1	209.5555	-0.0200	-3.4045	6.628E-04	3.692E-02
RPS9	2003.0047	0.0244	3.4046	6.625E-04	3.692E-02
DDX27	323.7471	-0.0237	-3.4005	6.727E-04	3.692E-02
SELT	764.6524	0.0281	3.3999	6.740E-04	3.692E-02
POLR3D	93.3525	-0.0202	-3.4118	6.453E-04	3.692E-02
KCNJ15	1412.8823	0.0334	3.3980	6.787E-04	3.696E-02
HAUS4	345.8233	0.0318	3.3904	6.980E-04	3.757E-02
RASGEF1B	170.1086	0.0224	3.3905	6.976E-04	3.757E-02
HIST1H4L	101.8307	0.0328	3.3880	7.042E-04	3.768E-02
CNIH4	230.1923	0.0264	3.3777	7.310E-04	3.867E-02
TMC6	1026.6305	-0.0231	-3.3779	7.303E-04	3.867E-02
TLR10	375.0385	0.0286	3.3757	7.363E-04	3.873E-02
FCGR2A	5605.8522	0.0305	3.3608	7.771E-04	3.954E-02
RASSF3	1355.9577	0.0270	3.3631	7.708E-04	3.954E-02
CKLF	104.8912	0.0306	3.3607	7.774E-04	3.954E-02
HDGFRP2	153.1896	-0.0224	-3.3645	7.668E-04	3.954E-02
VPS16	266.4049	-0.0146	-3.3647	7.662E-04	3.954E-02
SLC25A17	91.1802	-0.0203	-3.3636	7.695E-04	3.954E-02
LINC01128	177.3119	-0.0225	-3.3584	7.841E-04	3.967E-02
NOP2	149.5107	-0.0258	-3.3483	8.130E-04	3.975E-02
PPP3R1	1721.3182	0.0320	3.3524	8.012E-04	3.975E-02
DHX16	409.8418	-0.0175	-3.3479	8.143E-04	3.975E-02
MCM3	342.6960	-0.0241	-3.3465	8.185E-04	3.975E-02
PPP1R3B	1310.4863	0.0312	3.3500	8.082E-04	3.975E-02
UBXN2B	853.8443	0.0276	3.3464	8.188E-04	3.975E-02
SMARCA2	1318.7066	-0.0147	-3.3497	8.091E-04	3.975E-02
TXN	156.2888	0.0315	3.3459	8.202E-04	3.975E-02
VPRBP	350.6307	-0.0202	-3.3429	8.292E-04	3.998E-02
SAMD8	877.5832	0.0255	3.3410	8.349E-04	4.005E-02
NQO2	306.7456	0.0315	3.3337	8.569E-04	4.089E-02
CKAP5	435.1633	-0.0192	-3.3194	9.020E-04	4.094E-02
B3GAT3	96.7690	-0.0236	-3.3226	8.918E-04	4.094E-02
TBC1D10C	857.0018	-0.0157	-3.3215	8.953E-04	4.094E-02
ZFP36L1	520.8806	0.0288	3.3261	8.806E-04	4.094E-02

VPS18	245.7491	-0.0184	-3.3244	8.860E-04	4.094E-02
LMNB1	408.4101	0.0302	3.3220	8.938E-04	4.094E-02
MGAM2	161.5978	0.0328	3.3280	8.747E-04	4.094E-02
C9orf114	182.7995	-0.0221	-3.3230	8.905E-04	4.094E-02
MAN1B1	337.9157	-0.0237	-3.3219	8.940E-04	4.094E-02
HDAC6	283.7333	-0.0210	-3.3194	9.021E-04	4.094E-02
LONP1	228.3448	-0.0223	-3.3171	9.095E-04	4.107E-02
SMARCD2	599.6663	-0.0178	-3.3143	9.188E-04	4.129E-02
GALNT3	230.9563	0.0298	3.3063	9.452E-04	4.214E-02
TLR8	2144.9073	0.0308	3.3059	9.467E-04	4.214E-02
SLC4A1AP	212.9762	-0.0183	-3.2969	9.776E-04	4.278E-02
NLRC4	475.3910	0.0289	3.2979	9.741E-04	4.278E-02
SMIM14	230.2195	0.0230	3.2977	9.749E-04	4.278E-02
CA1	711.3247	0.0315	3.2963	9.796E-04	4.278E-02
IL27RA	323.1097	-0.0225	-3.2947	9.852E-04	4.282E-02
ACSL1	6647.6827	0.0323	3.2912	9.976E-04	4.316E-02
CLIC4	399.5928	0.0275	3.2885	1.007E-03	4.337E-02
LAX1	235.5768	-0.0208	-3.2848	1.020E-03	4.364E-02
FAM149B1	148.2723	0.0152	3.2842	1.023E-03	4.364E-02
GPATCH4	84.1741	-0.0241	-3.2799	1.038E-03	4.378E-02
AIDA	306.1466	0.0286	3.2759	1.053E-03	4.378E-02
PFKFB3	452.2728	0.0293	3.2786	1.043E-03	4.378E-02
ZAK	255.6290	0.0264	3.2755	1.055E-03	4.378E-02
UPP1	215.6865	0.0211	3.2779	1.046E-03	4.378E-02
RNF38	676.7639	0.0202	3.2789	1.042E-03	4.378E-02
PCSK7	811.0764	-0.0217	-3.2724	1.066E-03	4.408E-02
POMGNT1	131.8265	-0.0195	-3.2517	1.147E-03	4.452E-02
FCGR1A	114.0654	0.0318	3.2635	1.100E-03	4.452E-02
S100A6	867.2373	0.0248	3.2533	1.141E-03	4.452E-02
HSPA6	823.0560	0.0298	3.2646	1.096E-03	4.452E-02
BMS1	403.0865	-0.0226	-3.2557	1.131E-03	4.452E-02
PHRF1	362.5398	-0.0248	-3.2629	1.103E-03	4.452E-02
NUMA1	1610.0468	-0.0225	-3.2609	1.110E-03	4.452E-02
PLBD1	1563.3630	0.0284	3.2589	1.119E-03	4.452E-02
LAT	156.5532	-0.0239	-3.2635	1.101E-03	4.452E-02
EFTUD2	474.6533	-0.0204	-3.2665	1.089E-03	4.452E-02
USP39	316.0120	-0.0122	-3.2512	1.149E-03	4.452E-02
BID	760.1472	0.0250	3.2524	1.144E-03	4.452E-02
ATP6V0E1	942.6517	0.0233	3.2569	1.127E-03	4.452E-02
BAG4	226.1423	0.0242	3.2584	1.121E-03	4.452E-02
EFHC2	67.4761	0.0308	3.2608	1.111E-03	4.452E-02
CLIC2	91.1518	0.0318	3.2460	1.170E-03	4.515E-02
MYL12B	1520.8927	0.0256	3.2405	1.193E-03	4.531E-02
KREMEN1	221.5824	0.0315	3.2427	1.184E-03	4.531E-02
ZNF251	94.5587	-0.0229	-3.2405	1.193E-03	4.531E-02
CDK5RAP2	661.3612	-0.0178	-3.2402	1.194E-03	4.531E-02
ANTXRLP1	117.2362	-0.0305	-3.2382	1.203E-03	4.546E-02
TNRC6C-AS1	398.1363	-0.0263	-3.2368	1.209E-03	4.549E-02
UBE2B	765.8715	0.0298	3.2356	1.214E-03	4.551E-02
ADCK3	344.6657	-0.0222	-3.2316	1.231E-03	4.575E-02

KRI1	194.5480	-0.0249	-3.2306	1.236E-03	4.575E-02
USP19	546.9908	-0.0181	-3.2323	1.228E-03	4.575E-02
SART1	370.3734	-0.0224	-3.2261	1.255E-03	4.591E-02
ACER3	360.1435	0.0247	3.2271	1.251E-03	4.591E-02
SPTAN1	1336.1243	-0.0255	-3.2272	1.250E-03	4.591E-02
BCL11B	1538.2577	-0.0240	-3.2246	1.261E-03	4.597E-02
IFNAR1	1592.7745	0.0228	3.2215	1.275E-03	4.630E-02
MAP3K8	472.8969	0.0196	3.2192	1.286E-03	4.631E-02
SRA1	162.8698	0.0192	3.2199	1.282E-03	4.631E-02
FADS2	69.0598	-0.0317	-3.2130	1.314E-03	4.650E-02
ARPC3	1621.9386	0.0228	3.2164	1.298E-03	4.650E-02
CNIH1	127.3960	0.0264	3.2114	1.321E-03	4.650E-02
GLG1	1560.9191	-0.0212	-3.2154	1.302E-03	4.650E-02
IL10RB	770.9724	0.0221	3.2139	1.309E-03	4.650E-02
NUDCD3	554.1229	-0.0179	-3.2114	1.321E-03	4.650E-02
HIST2H2AC	1037.2112	0.0218	3.2043	1.354E-03	4.675E-02
URB2	187.9236	-0.0200	-3.2084	1.335E-03	4.675E-02
RASGRP2	1145.6281	-0.0189	-3.2064	1.344E-03	4.675E-02
ARHGEF18	1293.2802	-0.0210	-3.2036	1.357E-03	4.675E-02
RAB1A	615.5732	0.0207	3.2034	1.358E-03	4.675E-02
PUF60	367.8445	-0.0194	-3.2069	1.342E-03	4.675E-02
GCA	3769.3119	0.0316	3.1981	1.384E-03	4.744E-02
SUPT20H	642.3121	0.0208	3.1958	1.394E-03	4.763E-02
LSM10	86.7310	0.0250	3.1933	1.407E-03	4.788E-02
SLC16A6	255.5557	0.0255	3.1894	1.426E-03	4.800E-02
DAPK3	160.0749	-0.0214	-3.1898	1.424E-03	4.800E-02
ALDH1A1	170.1436	-0.0300	-3.1911	1.417E-03	4.800E-02
IFNGR2	1104.9346	0.0254	3.1873	1.436E-03	4.817E-02
CHD8	1230.9842	-0.0161	-3.1858	1.443E-03	4.824E-02
POLR2C	313.7424	0.0161	3.1838	1.454E-03	4.841E-02
DCLRE1C	542.2625	0.0146	3.1805	1.470E-03	4.847E-02
MTA2	672.4694	-0.0180	-3.1755	1.496E-03	4.847E-02
PLXNC1	5118.6268	0.0268	3.1776	1.485E-03	4.847E-02
IFI27	162.0368	-0.0205	-3.1752	1.497E-03	4.847E-02
LIG3	200.9274	-0.0189	-3.1753	1.497E-03	4.847E-02
FARSA	99.3406	-0.0230	-3.1820	1.463E-03	4.847E-02
PLCG1	1069.1708	-0.0250	-3.1795	1.475E-03	4.847E-02
LY86	138.7225	0.0256	3.1783	1.481E-03	4.847E-02
SNRNP200	1498.9976	-0.0209	-3.1734	1.507E-03	4.850E-02
MARCKS	564.4066	0.0311	3.1730	1.509E-03	4.850E-02
WDR45B	365.4102	-0.0124	-3.1699	1.525E-03	4.872E-02
HIGD1A	240.7011	0.0289	3.1696	1.526E-03	4.872E-02
SNX3	1617.9172	0.0304	3.1670	1.540E-03	4.901E-02
MIR4435-2HG	87.0635	0.0274	3.1643	1.555E-03	4.913E-02
GRAMD4	281.5459	-0.0211	-3.1650	1.551E-03	4.913E-02
PICALM	5663.2714	0.0262	3.1624	1.565E-03	4.927E-02
TLR1	3230.8812	0.0300	3.1614	1.570E-03	4.928E-02
TRAF6	659.2085	0.0175	3.1599	1.578E-03	4.937E-02

**Adjusted for age, gender, race, and pubertal status Base Mean: mean of normalized counts taken over all samples Beta: log_2 fold change estimate Stat: Wald test statistic per unit change of the phenotype as a continuous variable P-value: p-value corresponding to stat FDR: false discovery rate*

Table S2: All significant BMI-related differentially expressed genes associated with cardiometabolic phenotypes

Gene	Base Mean	Log_2 Fold Change	Stat	P value	FDR*
**FMD**					
ZAP70	890.2690	-0.0340	-4.6315	3.631E-06	1.075E-03
DRAM1	140.1020	0.0392	4.4123	1.023E-05	1.514E-03
CLIC4	415.7897	0.0351	4.0152	5.941E-05	4.412E-03
ANTXRLP1	121.1113	-0.0397	-3.9632	7.396E-05	4.412E-03
TMBIM4	856.0843	0.0329	3.8785	1.051E-04	4.412E-03
RAB1A	644.7973	0.0264	3.8774	1.056E-04	4.412E-03
NDUFB6	144.2577	0.0310	3.8666	1.104E-04	4.412E-03
MPI	142.4501	-0.0255	-3.8477	1.193E-04	4.412E-03
SCYL2	758.3485	0.0287	3.7723	1.617E-04	5.319E-03
PLCG1	1098.8306	-0.0312	-3.6980	2.173E-04	6.272E-03
WDR6	518.6541	-0.0303	-3.6802	2.331E-04	6.272E-03
SPTAN1	1366.8841	-0.0300	-3.5931	3.267E-04	8.059E-03
SUPT5H	341.4814	-0.0289	-3.5636	3.658E-04	8.298E-03
PCSK7	833.6786	-0.0251	-3.5451	3.925E-04	8.298E-03
DAPP1	1295.4082	0.0312	3.4891	4.847E-04	9.327E-03
TMC6	1057.4109	-0.0259	-3.4785	5.042E-04	9.327E-03
BAG4	235.8697	0.0276	3.4420	5.773E-04	1.005E-02
DEGS1	496.6802	0.0223	3.3871	7.063E-04	1.050E-02
NAIP	682.3777	0.0346	3.3824	7.185E-04	1.050E-02
TNFSF13B	954.7918	0.0353	3.3769	7.330E-04	1.050E-02
SEC62	1766.4547	0.0347	3.3724	7.453E-04	1.050E-02
GCA	4020.0211	0.0353	3.3350	8.530E-04	1.148E-02
TLR1	3415.6981	0.0336	3.3099	9.334E-04	1.193E-02
SAMD8	919.9679	0.0274	3.2898	1.003E-03	1.193E-02
GK	1123.3241	0.0334	3.2884	1.008E-03	1.193E-02
SERPINB1	1120.2462	0.0290	3.2394	1.198E-03	1.345E-02
NUDCD3	573.1694	-0.0196	-3.2205	1.280E-03	1.345E-02
C9orf72	1823.5843	0.0334	3.2132	1.313E-03	1.345E-02
ESYT1	1184.1542	-0.0242	-3.2112	1.322E-03	1.345E-02
CDK5RAP2	686.2534	-0.0193	-3.1997	1.375E-03	1.345E-02
LARS2	217.5633	-0.0180	-3.1928	1.409E-03	1.345E-02
BID	797.5867	0.0258	3.1555	1.602E-03	1.482E-02
PLBD1	1641.4239	0.0296	3.1463	1.654E-03	1.483E-02
THRA	83.8376	-0.0288	-3.1269	1.766E-03	1.538E-02
EIF2B5	368.8208	-0.0182	-3.1179	1.821E-03	1.540E-02
PICALM	5951.1154	0.0277	3.0806	2.066E-03	1.699E-02
LAPTM4A	315.4178	0.0225	3.0604	2.211E-03	1.768E-02
S100A9	18758.5775	0.0320	3.0323	2.427E-03	1.890E-02
NLRC4	501.0787	0.0285	3.0138	2.580E-03	1.958E-02
PHRF1	368.9176	-0.0243	-3.0029	2.675E-03	1.972E-02
LIG3	207.8538	-0.0192	-2.9893	2.796E-03	1.972E-02
NUMA1	1654.7434	-0.0225	-2.9885	2.804E-03	1.972E-02
KPNA4	1252.7830	0.0164	2.9655	3.022E-03	1.972E-02
TMEM59	1082.5722	0.0241	2.9601	3.076E-03	1.972E-02
TNRC6C-AS1	410.6954	-0.0266	-2.9595	3.081E-03	1.972E-02
UBE2B	804.6680	0.0297	2.9582	3.095E-03	1.972E-02

SRGN	7883.0991	0.0309	2.9471	3.208E-03	1.972E-02
ACTR10	300.6824	0.0226	2.9422	3.258E-03	1.972E-02
KCMF1	670.9463	0.0147	2.9416	3.265E-03	1.972E-02
KRI1	200.4503	-0.0239	-2.9323	3.364E-03	1.992E-02
LILRA5	1189.3658	0.0308	2.9077	3.642E-03	2.093E-02
CLIC2	94.6735	0.0306	2.8989	3.745E-03	2.093E-02
RASGRP2	1178.8988	-0.0195	-2.8986	3.748E-03	2.093E-02
MCCC1	218.7788	-0.0169	-2.8866	3.894E-03	2.120E-02
ZAK	268.7581	0.0242	2.8829	3.940E-03	2.120E-02
APEH	374.5665	-0.0139	-2.8403	4.507E-03	2.315E-02
CLIC1	2009.2155	0.0267	2.8400	4.512E-03	2.315E-02
TLR8	2260.4929	0.0285	2.8383	4.535E-03	2.315E-02
SELT	798.2869	0.0261	2.8223	4.769E-03	2.392E-02
HIGD1A	251.7157	0.0283	2.8136	4.899E-03	2.417E-02
CACTIN	139.6416	-0.0215	-2.7978	5.144E-03	2.459E-02
POLG	758.8821	-0.0155	-2.7954	5.184E-03	2.459E-02
ANXA3	255.0251	0.0291	2.7923	5.234E-03	2.459E-02
SULT1B1	320.7150	0.0289	2.7663	5.670E-03	2.622E-02
GPR160	206.4906	0.0259	2.7506	5.949E-03	2.660E-02
RIT1	660.5902	0.0226	2.7498	5.963E-03	2.660E-02
ATP8B2	1148.0763	-0.0232	-2.7467	6.020E-03	2.660E-02
MAP3K8	493.0291	0.0186	2.7299	6.336E-03	2.758E-02
METTL9	1019.6865	0.0248	2.7100	6.729E-03	2.886E-02
IFNGR2	1159.2305	0.0234	2.7022	6.887E-03	2.907E-02
WSB1	1261.2350	0.0218	2.6975	6.986E-03	2.907E-02
CKLF	110.6015	0.0266	2.6897	7.152E-03	2.907E-02
ARHGEF18	1329.4074	-0.0202	-2.6889	7.169E-03	2.907E-02
COX7A2L	309.9946	0.0236	2.6728	7.522E-03	3.009E-02
LONP1	235.3224	-0.0198	-2.6604	7.804E-03	3.080E-02
CYSTM1	167.9569	0.0275	2.6398	8.295E-03	3.205E-02
TNIK	597.4195	-0.0190	-2.6381	8.338E-03	3.205E-02
MS4A6A	920.8703	0.0248	2.6176	8.856E-03	3.348E-02
TP53BP1	458.6389	-0.0177	-2.6119	9.005E-03	3.348E-02
NAMPT	13617.3342	0.0275	2.6078	9.113E-03	3.348E-02
IFNAR1	1670.4073	0.0201	2.6059	9.163E-03	3.348E-02
LYZ	28178.4952	0.0225	2.5973	9.397E-03	3.392E-02
PFKP	254.4464	-0.0206	-2.5888	9.632E-03	3.399E-02
GLG1	1606.3066	-0.0192	-2.5882	9.647E-03	3.399E-02
ATP6V0E1	981.5998	0.0206	2.5731	1.008E-02	3.505E-02
VPRBP	362.2638	-0.0174	-2.5696	1.018E-02	3.505E-02
LETM1	209.2982	-0.0177	-2.5615	1.042E-02	3.524E-02
MARCKS	600.5288	0.0270	2.5596	1.048E-02	3.524E-02
HCAR2	264.2774	0.0267	2.5552	1.061E-02	3.530E-02
TLR5	266.0109	0.0261	2.5456	1.091E-02	3.584E-02
ZNF251	97.5193	-0.0199	-2.5420	1.102E-02	3.584E-02
ARPC3	1687.9305	0.0208	2.5383	1.114E-02	3.584E-02
FCGR2A	5887.7891	0.0248	2.5254	1.156E-02	3.678E-02
HSPA6	862.4797	0.0245	2.4999	1.242E-02	3.912E-02
PPP1R3B	1379.4681	0.0248	2.4921	1.270E-02	3.937E-02
EHMT2	157.4423	-0.0216	-2.4902	1.277E-02	3.937E-02

LINC01128	182.8392	-0.0185	-2.4800	1.314E-02	4.009E-02
POMGNT1	135.8941	-0.0166	-2.4630	1.378E-02	4.162E-02
LAX1	242.9219	-0.0180	-2.4515	1.423E-02	4.254E-02
ELAC2	387.4854	-0.0150	-2.4424	1.459E-02	4.319E-02
FARSA	101.4788	-0.0188	-2.4142	1.577E-02	4.622E-02
URB2	194.1059	-0.0169	-2.4099	1.596E-02	4.631E-02
MCM3	353.1588	-0.0188	-2.4042	1.621E-02	4.649E-02
CISD2	358.2423	0.0254	2.4007	1.636E-02	4.649E-02
CHD6	1146.2616	-0.0152	-2.3954	1.660E-02	4.649E-02
PWP2	245.2119	-0.0178	-2.3939	1.667E-02	4.649E-02
SNRNP200	1539.4452	-0.0179	-2.3895	1.687E-02	4.649E-02
RASA3	1155.6168	-0.0195	-2.3875	1.696E-02	4.649E-02
SLC16A6	269.5845	0.0212	2.3826	1.719E-02	4.669E-02
PLXNC1	5380.3536	0.0218	2.3695	1.781E-02	4.794E-02
PFKL	534.3045	-0.0175	-2.3569	1.843E-02	4.823E-02
HDAC6	291.8585	-0.0165	-2.3561	1.847E-02	4.823E-02
ACER3	378.6559	0.0204	2.3559	1.848E-02	4.823E-02
S100A6	902.9197	0.0203	2.3540	1.857E-02	4.823E-02
DUSP3	341.9312	0.0180	2.3383	1.937E-02	4.969E-02
CD58	385.3926	0.0207	2.3331	1.964E-02	4.969E-02
TRAF6	688.2301	0.0139	2.3324	1.968E-02	4.969E-02
TMEM165	537.3586	0.0211	2.3299	1.981E-02	4.969E-02
CARKD	188.2123	-0.0177	-2.3251	2.007E-02	4.991E-02
**Leptin**					
KREMEN1	238.0802	0.0238	4.3328	1.472E-05	4.358E-03
SRPK1	805.7845	0.0194	4.1526	3.288E-05	4.866E-03
PXK	383.5948	0.0135	3.9318	8.431E-05	6.300E-03
PLXNC1	5894.1825	0.0190	3.9295	8.513E-05	6.300E-03
DOCK4	485.7420	0.0212	3.8352	1.255E-04	7.428E-03
TLR5	284.1021	0.0203	3.7234	1.966E-04	9.697E-03
FCAR	267.4101	0.0197	3.6285	2.851E-04	1.062E-02
F5	573.5343	0.0196	3.6064	3.104E-04	1.062E-02
GK	1231.5137	0.0193	3.5466	3.903E-04	1.062E-02
SLC37A3	340.4001	0.0159	3.5355	4.069E-04	1.062E-02
MAPK14	2559.9372	0.0160	3.5210	4.300E-04	1.062E-02
FCGR2A	6223.3225	0.0179	3.5002	4.648E-04	1.062E-02
SULT1B1	352.8954	0.0192	3.4806	5.003E-04	1.062E-02
CD55	2302.5961	0.0149	3.4706	5.192E-04	1.062E-02
ENC1	266.7449	0.0184	3.4610	5.383E-04	1.062E-02
ITGB7	810.7305	-0.0158	-3.4377	5.867E-04	1.085E-02
ACSL1	7139.3703	0.0186	3.3794	7.266E-04	1.265E-02
ARL11	576.4557	0.0155	3.3093	9.353E-04	1.517E-02
GCA	4328.1368	0.0183	3.2979	9.740E-04	1.517E-02
MGAM2	186.8399	0.0181	3.2712	1.071E-03	1.525E-02
PPP1R3B	1462.0762	0.0172	3.2682	1.082E-03	1.525E-02
IFNGR2	1269.0633	0.0149	3.2241	1.264E-03	1.700E-02
IFNAR1	1828.0383	0.0134	3.1729	1.509E-03	1.899E-02
IFI27	90.6295	-0.0147	-3.1671	1.540E-03	1.899E-02
SLC16A6	304.4865	0.0150	3.1460	1.655E-03	1.956E-02
PROK2	738.4741	0.0174	3.1333	1.729E-03	1.956E-02

AQP9	3797.1378	0.0167	3.1216	1.799E-03	1.956E-02
ADM	279.6332	0.0171	3.1043	1.908E-03	1.956E-02
KCNJ15	1616.5936	0.0172	3.1023	1.921E-03	1.956E-02
SOCS3	349.7996	0.0168	3.0928	1.983E-03	1.956E-02
NAMPT	14091.5587	0.0169	3.0764	2.095E-03	2.001E-02
HSPA6	917.7216	0.0158	3.0356	2.401E-03	2.221E-02
IL1B	354.7358	0.0161	3.0197	2.530E-03	2.269E-02
DEGS1	548.5614	0.0095	2.9869	2.818E-03	2.454E-02
HCAR2	279.0772	0.0164	2.9690	2.988E-03	2.527E-02
C9orf72	2031.4993	0.0164	2.9566	3.111E-03	2.558E-02
TMCO3	400.7916	0.0116	2.9372	3.311E-03	2.649E-02
CYSTM1	177.2628	0.0162	2.9247	3.448E-03	2.686E-02
DDX27	377.8719	-0.0120	-2.9108	3.605E-03	2.736E-02
IL4R	1717.2587	0.0125	2.8992	3.741E-03	2.769E-02
UBXN2B	980.0951	0.0142	2.8912	3.838E-03	2.771E-02
RGL4	154.6686	0.0137	2.8834	3.934E-03	2.772E-02
MEF2A	847.1092	0.0124	2.8647	4.174E-03	2.831E-02
NFKBIZ	1180.7371	0.0130	2.8622	4.208E-03	2.831E-02
LILRA5	1226.8854	0.0157	2.8326	4.617E-03	2.882E-02
DPY19L3	386.8050	0.0127	2.8312	4.638E-03	2.882E-02
VCPKMT	268.8749	0.0133	2.8303	4.650E-03	2.882E-02
NFIL3	266.1845	0.0146	2.8287	4.673E-03	2.882E-02
TMBIM4	967.4486	0.0131	2.8000	5.110E-03	3.031E-02
WSB1	1421.9734	0.0120	2.7953	5.185E-03	3.031E-02
TLR8	2468.0483	0.0151	2.7930	5.223E-03	3.031E-02
GPR160	235.3555	0.0143	2.7802	5.433E-03	3.092E-02
NMT2	359.4494	-0.0118	-2.7467	6.019E-03	3.362E-02
CARKD	214.3463	-0.0112	-2.7256	6.418E-03	3.518E-02
CKAP5	513.7246	-0.0089	-2.7136	6.655E-03	3.582E-02
ST3GAL4	147.0653	0.0144	2.6976	6.983E-03	3.630E-02
TLR1	3720.2194	0.0147	2.6973	6.990E-03	3.630E-02
EHMT2	177.8124	-0.0129	-2.6754	7.463E-03	3.809E-02
PLCG1	1243.9924	-0.0128	-2.6623	7.762E-03	3.885E-02
KCMF1	758.2840	0.0073	2.6574	7.874E-03	3.885E-02
BMS1	478.7794	-0.0112	-2.6491	8.071E-03	3.916E-02
ZAK	306.4864	0.0126	2.6029	9.244E-03	4.337E-02
UPP1	249.2912	0.0102	2.5940	9.486E-03	4.337E-02
SPTAN1	1558.8412	-0.0119	-2.5911	9.567E-03	4.337E-02
DHRS13	188.7412	0.0135	2.5909	9.573E-03	4.337E-02
MAP3K8	552.6267	0.0102	2.5806	9.863E-03	4.337E-02
ANXA3	249.6743	0.0139	2.5788	9.914E-03	4.337E-02
PICALM	6647.4994	0.0129	2.5748	1.003E-02	4.337E-02
SAMD8	1034.0056	0.0117	2.5684	1.022E-02	4.337E-02
HCAR3	344.1442	0.0142	2.5671	1.026E-02	4.337E-02
TNFSF13B	1016.3749	0.0141	2.5525	1.069E-02	4.458E-02
TMEM260	527.6265	0.0101	2.5275	1.149E-02	4.722E-02
SEC62	2088.5796	0.0139	2.5099	1.208E-02	4.896E-02
EBLN2	125.8482	0.0106	2.5042	1.227E-02	4.909E-02
**CRP**					
TLR5	338.2794	4.5880E-05	3.9804	6.880E-05	1.1400E-02

JAZF1	1125.0407	4.6157E-05	3.9062	9.375E-05	1.1400E-02
ITGB7	882.4707	-3.5154E-05	-3.8150	1.362E-04	1.1400E-02
MEF2A	962.7162	3.2388E-05	3.7512	1.760E-04	1.1400E-02
DOCK4	569.9893	4.3571E-05	3.7220	1.976E-04	1.1400E-02
FCGR1A	162.3060	4.2510E-05	3.6461	2.662E-04	1.1400E-02
MGAM2	212.2949	4.3333E-05	3.6429	2.696E-04	1.1400E-02
FCAR	303.7247	4.0630E-05	3.5366	4.054E-04	1.1458E-02
ENC1	301.8799	3.9964E-05	3.5347	4.083E-04	1.1458E-02
FCGR1B	145.6515	4.1875E-05	3.5199	4.317E-04	1.1458E-02
PRPF31	245.8076	-2.7672E-05	-3.5187	4.337E-04	1.1458E-02
TCF7L2	118.1338	3.8829E-05	3.5004	4.645E-04	1.1458E-02
WSB1	1628.5150	2.9987E-05	3.4578	5.446E-04	1.1736E-02
GK	1464.6233	3.9432E-05	3.4527	5.551E-04	1.1736E-02
PWP2	298.3533	-2.7067E-05	-3.3761	7.351E-04	1.3878E-02
AIDA	440.0362	3.6034E-05	3.3706	7.502E-04	1.3878E-02
LEPROT	551.6261	2.9422E-05	3.3367	8.479E-04	1.4764E-02
LAX1	302.8443	-2.6152E-05	-3.2265	1.253E-03	2.0609E-02
ACER3	494.3961	2.9847E-05	3.1397	1.691E-03	2.6351E-02
NAIP	893.2463	3.6304E-05	3.1187	1.817E-03	2.6888E-02
SLC16A6	345.6264	2.9173E-05	3.0214	2.516E-03	3.5470E-02
TNFSF13B	1264.6375	3.4987E-05	2.9936	2.757E-03	3.7089E-02
SERPINB1	1440.9944	3.0744E-05	2.9701	2.977E-03	3.8130E-02
CISD2	499.4121	3.5188E-05	2.9585	3.092E-03	3.8130E-02
C9orf72	2439.4325	3.3614E-05	2.9285	3.405E-03	4.0321E-02
CACTIN	163.8464	-2.1746E-05	-2.9071	3.648E-03	4.1536E-02
IL1B	411.6949	3.3140E-05	2.8788	3.992E-03	4.3760E-02
THYN1	192.0114	-1.6487E-05	-2.8266	4.704E-03	4.9274E-02
UBE2B	1072.3835	3.0769E-05	2.8183	4.827E-03	4.9274E-02

**Adjusted for age, gender, pubertal status, and percent total body fat*

Base Mean: mean of normalized counts taken over all samples
Log_2 Fold Change: log_2 fold change estimate
Stat: Wald test statistic per unit change of the phenotype as a continuous variable
P-value: p-value corresponding to stat
FDR: false discovery rate

Table S3: Top 20 differentially expressed genes associated with FMD%			
**Gene**	**Name**	**Category**	**FDR***
ZAP70	zeta chain of T-cell receptor associated protein kinase 70	immune function (T cell signaling)	1.075E-03
DRAM1	DNA damage regulated autophagy modulator 1	general cellular function (oncogenic regulation)	1.514E-03
CLIC4	chloride intracellular channel 4	general cellular function (ionic homeostasis)	4.412E-03
ANTXRLP1	anthrax toxin receptor-like pseudogene	--	4.412E-03
TMBIM4	transmembrane BAX inhibitor motif containing 4	general cellular function (mediating apoptosis, Ca ion regulation)	4.412E-03
RAB1A	RAB1A, member RAS oncogene family	general cellular function (trafficking)	4.412E-03
NDUFB6	NADH:ubiquinone oxidoreductase subunit B6	metabolic effector (mitochondrial component)	4.412E-03
MPI	mannose phosphate isomerase	metabolic effector	4.412E-03
SCYL2	SCY1 like pseudokinase 2	general cellular function (trafficking)	5.319E-03
PLCG1	phospholipase C gamma 1	general cellular function (intracellular cell signaling)	6.272E-03
WDR6	WD repeat domain 6	general cellular function (cell cycle)	6.272E-03
SPTAN1	spectrin alpha, non-erythrocytic 1	general cellular function (DNA repair, cell cycling, structural)	8.059E-03
SUPT5H	SPT5 homolog, DSIF elongation factor subunit	general cellular function (post-translational mRNA processing)<br>immune function (relevant to HIV infectivity)	8.298E-03
PCSK7	proprotein convertase subtilisin/kexin type 7	general cellular function (trafficking, Fe metabolism)<br>potential immune function (HIV envelope, B-cell)	8.298E-03
DAPP1	dual adaptor of phosphotyrosine and 3-phosphoinositides 1	immune function	9.327E-03
TMC6	transmembrane channel like 6	general cellular function (ER membrane channel)	9.327E-03
BAG4	BCL2 associated athanogene 4	general cellular growth (apoptosis)	1.005E-02
DEGS1	delta 4-desaturase, sphingolipid 1	metabolic effector (fatty acid desaturation)	1.050E-02
NAIP	NLR family apoptosis inhibitory protein	inflammatory function (inflammasome; apoptosis)	1.050E-02
TNFSF13B	TNF superfamily member 13b	Immune/inflammatory signaling	1.050E-02

*Adjusted for age, gender, pubertal status, and percent total body fat

FDR: false discovery rate

Table S4: Top 20 differentially expressed genes associated with leptin

Gene	Name	Category	FDR*
KREMEN1	kringle containing transmembrane protein 1	general cellular function (signaling)	4.358E-03
SRPK1	SRSF protein kinase 1	general cell transcriptional regulation	4.866E-03
PXK	PX domain containing serine/threonine kinase like	integumentary effector	6.300E-03
PLXNC1	plexin C1	immune function<br>general cellular function (motility)	6.300E-03
DOCK4	dedicator of cytokinesis 4	general structural/adhesion function	7.428E-03
TLR5	toll like receptor 5	Innate immune signaling	9.697E-03
FCAR	Fc receptor of IgA	immune function (humoral effector, opsonization)	1.062E-02
F5	coagulation factor V	vascular function (coagulation)	1.062E-02
GK	glycerol kinase	metabolic effector	1.062E-02
SLC37A3	solute carrier family 37 member 3	potential metabolic effector (regulator of adipose tissue)	1.062E-02
MAPK14	mitogen-activated protein kinase 14	general cellular function<br>immune function (responds to inflammation)	1.062E-02
FCGR2A	Fc fragment of IgG receptor IIa	immune function (pro-inflammatory humoral effector)	1.062E-02
SULT1B1	sulfotransferase family 1B member 1	general cellular function (enzyme)	1.062E-02
CD55	cluster of differentiation 55	immune function (regulator of complement-driven cellular damage)	1.062E-02
ENC1	ectodermal-neural cortex 1	general structural function<br>oxidative stress responder	1.062E-02
ITGB7	integrin beta 7	immune function (adhesion)	1.085E-02
ACSL1	acyl-CoA synthetase long chain family member 1	general metabolic function (lipid/fatty acid catabolism)	1.265E-02
ARL11	ADP ribosylation factor like GTPase 11	general cell cycle function (intracellular signaling; apoptosis inducer)	1.517E-02
GCA	grancalcin	immune function (ionic homeostasis in immune cells)	1.517E-02
MGAM2	maltase-glucoamylase 2 (putative)	general metabolic function (glycogenolysis)	1.525E-02

** Adjusted for age, gender, pubertal status, and percent total body fat*

FDR: false discovery rate

Table S5: Top 20 differentially expressed genes associated with CRP

Gene	Name	Category	FDR*
TLR5	toll like receptor 5	innate immune signaling	1.140E-02
JAZF1	JAZF zinc finger 1	transcriptional repressor	1.140E-02
ITGB7	integrin beta 7	cell adhesion (immune cells, endothelial cells)	1.140E-02
MEF2A	myocyte enhancer factor 2A	muscular effector	1.140E-02
DOCK4	dedicator of cytokinesis 4	general structural/adhesion function	1.140E-02
FCGR1A	Fc receptor of IgG receptor	immune function (opsonization; pro-inflammatory activation)	1.140E-02
MGAM2	maltase-glucoamylase 2 (putative)	general metabolic function (glycogenolysis)	1.140E-02
FCAR	Fc receptor of IgA	immune function (opsonization; pro-inflammatory activation)	1.146E-02
ENC1	ectodermal-neural cortex 1	general structural function<br>oxidative stress responder	1.146E-02
FCGR1B	Fc receptor 1B of IgG	immune function (opsonization; pro-inflammatory activation)	1.146E-02
PRPF31	pre-mRNA processing factor 31	post-transcriptional modifications	1.146E-02
TCF7L2	transcription factor 7 like 2	metabolic effector (serum glucose metabolism)	1.146E-02
WSB1	WD repeat and SOCS box containing 1	general cellular functions	1.174E-02
GK	glycerol kinase	metabolic effector	1.174E-02
PWP2	periodic tryptopha protein 2	general cellular function	1.388E-02
AIDA	axin interactor, dorsalization associated	structural mediator (embryogenesis)	1.388E-02
LEPROT	leptin receptor overlapping transcript	general cellular function (signaling, trafficking)	1.476E-02
LAX1	lymphocyte transmembrane adaptor 1	immune function (T-Cell down-regulator)	2.061E-02
ACER3	alkaline ceramidase 3	cellular metabolism regulator	2.635E-02
NAIP	NLR family apoptosis inhibitory protein	inflammatory response (inflammasome; apoptosis)	2.689E-02

** Adjusted for age, gender, pubertal status, and percent total body fat*

FDR: false discovery rate
